# Surgical management and clinical outcome of cervical, thoracic and thoracolumbar spinal tuberculosis in a middle-European adult population

**DOI:** 10.1038/s41598-023-34178-9

**Published:** 2023-04-28

**Authors:** Fatma Kilinc, Matthias Setzer, Bedjan Behmanesh, Daniel Jussen, Florian Geßler, Vincent Prinz, Marcus Czabanka

**Affiliations:** 1 Department of Neurosurgery, Goethe University Hospital, Schleusenweg 2-16, 60528 Frankfurt am Main, Germany; 2 Department of Neurosurgery, University Medicine of Rostock, Rostock, Germany

**Keywords:** Diagnosis, Disease prevention, Quality of life

## Abstract

Spinal tuberculosis is due to globalization no longer a disease limited to developing nations. It remains in Germany a rarity and still a difficult diagnosis. Here we analyzed patients with spinal tuberculosis treated at our neurosurgical department. According to the infected anatomic segment, patients were assigned in one of three groups. Surgery was performed when neurological deficit due to mechanical compression, deformity, instability, severe pain, necrotic bone or failure to respond to anti-tuberculous treatment were observed. We identified 34 patients with spinal tuberculosis who underwent surgical treatment. In the cervical spinal tuberculosis group, there were 15 cases (46.9%) In most cases treatment consisted of spinal instrumentation. In the thoracic group, 10 cases (29.4%) were observed. The treatment was performed by dorsolateral spinal instrumentation. For the thoracolumbar group, 9 cases (26.4%) were observed. In most cases dorsolateral spinal instrumentation was performed. One patient in the first group and one patient in the third group relapsed after operation. A second surgery was necessary. Patients with chronic back pain, immigration background and/or neurological deficit spinal TB should be considered as a differential diagnosis. Combined surgical intervention and medical treatment is associated with a favorable outcome.

## Introduction

Tuberculosis (TB) caused by Mycobacterium tuberculosis is one of the oldest disease in the world. It causes more than 2 million deaths annually worldwide^1,2^.

Extra-pulmonary TB occurs in 15–20% of TB-patients^3^. A frequently encountered extrapulmonary form of this disease is spinal tuberculosis, also known as Pott´s disease, which can cause serious clinical condition with severe deformity and neurological complications like complete paraplegia or tetraplegia^4,5^.

Despite a marked improvement in the socioeconomic status of many countries and the availability of effective antitubercular drugs, the impact of the acquired immunodeficiency syndrome (AIDS) pandemic and immigration have increased the rate of this disease^6^.

The diagnosis is often challenging and can be delayed. Early diagnosis and treatment are necessary to prevent permanent neurological disability and to minimize spinal deformity^7^.

The majority of cases occur in population-dense developing countries like Africa or India, however due to globalization and travelling it is no longer a disease limited to developing nations^4,10^. Most cases in the western world of spinal tuberculosis are seen primarily in immigrants form endemic countries. While in endemic countries, Pott´s disease is more common in children and younger adults, in developed Western and Middle East countries adults are affected^4,10,11^. The upper lumbar and lower thoracic region of vertebral column is most frequently affected. Characteristically for spinal tuberculosis is destruction of the intervertebral disk space and the adjacent vertebral bodies. Collapse of the spinal elements and anterior wedging leading to the characteristic angulation and gibbus formation^9^. Surgical treatment is considered in cases of severe spinal instability or progressive neurological symptoms with evidence of cord compression or deformation^8^.

So far spinal tuberculosis remains in Germany a rarity and still a difficult diagnosis. Here we analysed the surgical treatment and clinical outcomes of patients with a cervical, thoracic, and thoracolumbar spinal tuberculosis in a middle European metropolitan adult population.

## Methods

This study was performed at the neurosurgical department of Goethe-University Frankfurt. Patients with spinal tuberculosis between 2014 and 2022 were retrospectively analyzed. Aim of this study was to analyse the surgical treatment and clinical outcome of patients with a cervical, thoracic, and thoracolumbar spinal tuberculosis in a middle Europe metropolitan city.

All patient data were collected from the electronic patient records. Spinal TB was diagnosed in patients who had symptoms and signs compatible with spinal TB. For diagnose spinal tuberculosis the detection of mycobacterium tuberculosis or acid-fast bacilli (AFB) was necessary.

The diagnosis was made in the presence of clinical manifestations and radiological as well as haematological examination or by preoperatively tissue sampling by CT-guided biopsy. Diagnosis could also established by radiological manifestations (CT scan and/or MRI) and/or clinical features that correlated with spinal TB and had a good response to anti-TB therapy^12,13^.

All included patients were sorted into 3 groups according to the affected region of vertebral column. According to the infected anatomic segment, patients were assigned in the cervical, thoracic or thoracolumbar group.

All patients were evaluated clinically using the visual analog scale (VAS). Patient characteristics affected spinal levels, initial symptoms, duration of symptoms until surgery, American Spinal Injury Association (ASIA) impairment scale for assessment of neurological status as well as the surgical procedure and outcome were analyzed.

Surgery was performed when neurological deficit due to mechanical compression, deformity, instability, severe pain, necrotic bone or failure to respond anti-tuberculous treatment were observed. The surgical procedure was defined as dorsal, dorsoventral or debridment with instrumentation and/or vertebral curettage. If dorsal procedure was necessary, posterior internal fixation was performed. For dorsoventral surgical procedure also a vertebral body replacement was done. If debridement of infected vertebrae was necessary disc material and fusion were performed via an anterior approach.

Long term follow-up as well as complications related to surgery were recorded and analyzed.

### Ethics statement

This study was approved by the local ethics committee of the Goethe University Frankfurt am Main, the methods were carried out in accordance with the relevant guidelines and regulations. Informed consent was obtained in each case.

## Results

We identified a total of 34 patients with spinal tuberculosis who underwent surgical treatment in our department. All included patients had immigrant background. 23 patients (67.6%) were male, and 11 female (32.4%), with a mean age of 36.5 ± 17.1 years (range, 23–88 years.

In 23 cases (67.6%) CT-guided biopsy was already performed preoperatively to confirm the diagnosis of spinal tuberculosis. Of these 23 procedures 5 (21%) were inconclusive because of inadequate sample (40%), reactive tissue (40%) or normal tissue (20%). However diagnosis was confirmed for these patients by tissue obtained intraoperatively. In 87% of analyzed cases, positive evidence was also obtained from haematological examinations.

In most cases spinal tuberculosis was located cervical (44.2%) followed by thoracal (29.4). In five cases the location was lumbar (14.7%) an in two cases each (5.9%), the thoracolumbar junction (TH12/L1) and the lumbosacral junction (L5/S 1) were affected. These patients were summarized under Table 4. The demographics and clinical characteristics of the 34 patients with spinal TB are summarized in Table 1.

**Table 1:** Demographic and clinical data of patients with spinal tuberculosis (n = 34).

Variable	
Age (mean, yr)	36.5 ± 17.1
Sex, male:female	23:11
Follow up (mean, months)	37.1 ± 31.8
Duration of symptoms to diagnosis (mo)	5 ± 3
Laboratory findind on admission	
CRP	4.87 ± 4.41(mg/dl)
WBC	8.87 ± 3.19(/μl)
Symptoms	
Pain	24 (70.6)
Neurological deficits	9 (26.5)
ASIA D	8 (23.5)
ASIA C	1 (2.9)
Fever	10 (29.4)
Diagnosis	
CT-guided biopsy	18 (53)
Intraoperative tissue	32 (94)
Haemtological	30 (88)
Location of spinal TB	
Cervical	15 (44.2)
Thoracic	10 (29.4)
Thoracolumbar	2 (5.9)
Lumbar	5 (14.7)
Lumbosacral	2 (5.9)
Image findings	
Presence of abscess	9 (26.5)
Spinal kyphotic deformity	5 (14.7)
Collapse vertebra	18 (52.9)
Cord compression	4 (11.8)
Number of vertebra involved	2 ± 1
Surgical procedure	
Dorsal	21 (61.8)
Dorsoventral	11 (32.4)
Debridement	2 (5.9)
Relaps after surgery	2 (5.9)

In the cervical spinal tuberculosis group, there were 15 cases (46.9%) with a mean age of 31.6 ± 12 years. In 13 cases (86.7%) treatment consisted of dorsal spinal instrumentation. For two patient surgical debridement of the infected vertebrae, disc material and fusion were performed via an anterior approach (Table 2).

**Table 2:** Demographic and clinical data of patients with cervical spinal tuberculosis (n = 15).

Variable	
Age (mean, yr)	31.6 ± 12
Sex, male:female	11:4
Follow up (mean, months)	48.5 ± 42.8
Duration of symptoms to diagnosis (mo)	2 ± 1
Laboratory findind on admission	
CRP	3 ± 1.59(mg/dl)
WBC	8.45 ± 2.71(/ul)
Symptoms	
Pain	14 (93.3)
Neurological deficits	1 (6.7)
ASIA D	1 (6.7)
Fever	3 (20)
Image findings	
Presence of abscess	9 (26.5)
Spinal kyphotic deformity	2 (13.3)
Collapse vertebra	9 (60)
Cord compression	0 (0)
Number of vertebra involved	2 ± 1
Surgical procedure	
Dorsal instrumentation	13 (86.7)
Debridement	2 (13.4)
Relaps after surgery	1 (6.7)
Revision surgery	1 (6.7)

In the thoracic spinal tuberculosis group, there were 10 cases (29.4%) with a mean age of 37.7 ± 16 years. The treatment was performed by dorsolateral spinal instrumentation (Table 3).

**Table 3:** Demographic and clinical data of patients with thoracic spinal tuberculosis (n = 10).

Variable	
Age (mean, yr)	37.7 ± 16
Sex, male:female	7:3
Follow up (mean, months)	22.1 ± 19
Duration of symptoms to diagnosis (mo)	2 ± 1
Laboratory findind on admission	
CRP	7.1 ± 5.35(mg/dl)
WBC	10.14 ± 3.09(/ul)
Symptoms	
Pain	10 (100)
Neurological deficits	2 (20)
ASIA D	1 (10)
ASIA C	1 (10)
Fever	3 (30)
Image findings	
Presence of abscess	2 (20)
Spinal kyphotic deformity	6 (60)
Collapse vertebra	8 (80)
Cord compression	2 (20)
Number of vertebra involved	2 ± 1
Surgical treatment	
Dorsal instrumentation	5 (50)
Dorsolaterall	5 (50)
Relaps after surgery	0(0)
Revision surgery	0(0)

The thoracolumbar spinal tuberculosis group shows 9 cases (26.4%) with a mean age of 44.1 ± 21 years. In six cases dorsolateral spinal instrumentation and in three cases only spinal instrumentation was performed (Table 4).

**Table 4:** Demographic and clinical data of patients with thoracolumbar spinal tuberculosis (n = 9).

Variable	
Age (mean, yr)	44.1 ± 21
Sex, male:female	5:4
Follow up (mean, months)	35.1.5 ± 4.5
Duration of symptoms to diagnosis (mo)	9.33 ± 4.15
Laboratory findind on admission	
CRP	4.92 ± 4.78(mg/dl)
WBC	7.12 ± 2.98(/ul)
Symptoms	
Pain	9 (100)
Neurological deficits	6 (66.7)
ASIA D	6 (66.7)
Fever	1 (11.1)
Image findings	
Presence of abscess	2 (22.2)
Spinal kyphotic deformity	0 (0)
Collapse vertebra	3 (33.3)
Cord compression	5 (55.6)
Number of vertebra involved	1 ± 0
Surgical procedure	
Dorsal	3 (33.3)
Dorsolaterall	6 (66.7)
Relaps after surgery	1 (11.1)
Revision surgery	1 (11.1)

At the end of follow-up period (Table 5) the outcome for 34 patients were evaluated. The mean follow-up time was 37.1 ± 31.8 (range 6–96 months). All included patients had a favorable outcome and demonstrated a clinical healing of tuberculosis infection. Bony fusion was achieved in 94.1% with no loosening or breakage of internal fixation. In all three groups, the VAS was scored 8 weeks after operation and at the most recent follow-up. One patient in the first group and one patient in the third group relapsed after surgery. A second surgery was necessary. In eight cases (23.5%) neurological impairment (ASIA D) was described, which regressed completely (ASIA E). In the thoracic cohort in one case ASIA C was observed. In the last follow-up a complete regression was observed (ASIA E).

**Table 5:** Outcome at last follow.

	Preoperatively	Postoperatively
VAS	7.53 ± 2.5	1.91 ± 1.44
ASIA E	25 (73.5)	34 (100)
ASIA D	8 (23.5)	0 (0)
ASIA C	1 (2.9)	0 (0)

## Discussion

In this study we analyzed the clinical course and the long-term neurological outcome in patients with spinal tuberculosis who were treated in our neurosurgical department.

Although it has been estimated that extra-pulmonary TB occurs in 15–20% of TB-patients and the frequently encountered extrapulmonary form of this disease is spinal TB, the true incidence of spinal TB is still unknown^3^. Recent data describe a rise in the number of cases, also in western countries^1,2,14^. Due to globalization and travelling it is no longer a disease limited to developing nations. In western countries spinal TB is primarily seen in immigrants from developing countries^14^. This was confirmed in our cohort as well.

Despite a marked improvement in the socioeconomic status of many countries and the availability of effective antitubercular drugs, the impact of the acquired immunodeficiency syndrome (AIDS) pandemic and immigration have increased the rate of the disease^6^.

TB is generally curable with quadruble anit-TB chemotherapy, and it still remains to be determined whether there is a need for surgical intervention in spinal TB. The literature describes that neurological deficits can recover well on tuberculostatic drug therapy and spinal tuberculosis can be healed without surgical procedures^4,15^.

However, owing the additional problems encountered in spinal TB such as kyphotic angle and neurological deficits, several studies appealed for surgical intervention^16–18^. Recommendation for surgical treatment of spinal tuberculosis are also include treating pronounced stenoses, to create better recovery possibilities, faster pain reduction and to restore mobility and independence in daily life^4,17–20^. Surgical treatment allows to achieve early decompression of the cord, histological verification of the diagnosis and prompt relief of most of the patient´s symptoms. Furthermore it reduces the risk of maintained cord compression—even after successful medical treatment due to unexcised granuloma which is replaced by fibrous scar tissue^21,22^.

All patients who were included in our study were treated surgically. Reasons for surgical treatment was neurological deficit due to mechanical compression, deformity, instability, severe pain, necrotic bone or failure to respond anti-tuberculous treatment.

Park et al. reported surgery in conjuction with quadruple anti-TB chemotherapy with a favourable result. Su et al.^12,23^ also reported cases combined with surgical intervention tended to have more favourable outcomes.

In 5% of the analyzed cases, conservative therapy with anti-TB chemotherapy because of concomitant pulmonary TB was already started. In the remaining 5%, chemotherapy was started postoperatively after confirmation of the diagnosis. While neurological involvement was reported in literature in 23–76%, we found it in 27.5% of the cases^21,24,25^. All patients with neurological deficits had no more deficits at the last follow-up. Postoperatively also an impressive reduction in pain was described.

In comparison with previously published data, we found some similarities to our analyzed results. In literature the most common presenting symptoms of spinal TB were back pain^25–27^. In our study 70.6% of analysed patients described back pain^16,22,25^. The mean duration of symptoms prior to diagnosis is from 3–6 months^25,28^. In our cohort the mean duration of symptoms were 5 ± 3 months. Garg et al.^4^reported that a longer duration of symptoms to diagnosis was associated with poor outcome. In Frankfurt, which has good access to medical care the time from symptoms to diagnosis cervical and/or thoracic spinal tuberculosis were shorter than in other studies^23^. In return, the diagnosis of thoracolumbar spinal TB were longer (9.33 ± 4.15). Therefore in patients with back pain and immigration background clinicians should be more aware of the possibility of spinal TB. Fever is not common, with a reported rate of 31–45%^16,22,25^. We identified in ten cases (29.4%) fever.

Spinal TB develops mainly in adolescents and young adults in developing countries. In developed countries older adults are affected^28,29^. In our cohort, the mean age of spinal TB was 36.5 ± 17.1 years.

Traditionally, spinal TB has been reported to be most prevalent in the thoracic spine, however some studies have observed spinal TB to be more common in the lumbar spine^30,31^. Similarly, our study have shown that spinal TB occurs mainly in the thoracolumbar and lumbar spines.

## Conclusion

Patients with chronic back pain, immigration background and/or neurological deficit spinal TB should be considered as a differential diagnosis. Aggressive diagnosis of spinal TB is necessary and combined surgical intervention and medical treatment is associated with a good outcome.

### Limitation

This study is limited by its retrospective design. The study is also limited by the small number of patients. Treatment of tuberculosis is a long process. Therefore, it is necessary to assess the clinical course of patients with a long follow-up period.

## Data Availability

The datasets generated during and/or analyzed during the current study are available from the corresponding author on reasonable request.
